# Investigating the Effects of Meditation, Nature Sounds, and Music on the Patient Experience During Intravitreal Injections

**DOI:** 10.7759/cureus.89483

**Published:** 2025-08-06

**Authors:** Renae A Tessem, Alexander B Dillon, Boss Le, Justin Hanson, Maggie Hui, Lexy Anderson, Ami Tamhaney, Fei Yu, Moritz S Pettenkofer, Tara A McCannel

**Affiliations:** 1 Ophthalmology Department, David Geffen School of Medicine at the University of California, Los Angeles (UCLA), Los Angeles, USA; 2 Vitreoretinal Surgery Service, Kaiser Permanente, Vallejo, USA; 3 Retina Division, University of California, Los Angeles (UCLA) Jules Stein Eye Institute, Los Angeles, USA; 4 Biostatistics Division, University of California, Los Angeles (UCLA) Jules Stein Eye Institute, Los Angeles, USA; 5 Ophthalmic Oncology Center, University of California, Los Angeles (UCLA) Jules Stein Eye Institute, Los Angeles, USA

**Keywords:** anxiety, intravitreal injection, meditation, music, pain, patient satisfaction

## Abstract

Introduction: The purpose of this study was to evaluate the effects of three brief interventions (spoken/guided meditation, nature sounds, or music) on patients' subjective experience with intravitreal injections (IVIs).

Methods: A total of 121 consecutive patients were randomized into four cohorts: spoken/guided meditation (n = 31), nature sounds (n = 30), music (classical or jazz, n = 30), and control (n = 30). Subjective anxiety levels were recorded prior to and following a three- to five-minute intervention in the first three study groups as well as following the IVI. Controls did not participate in an intervention prior to the injection. Subjective pain with the injection was recorded, as was patient satisfaction with the injection experience.

Results: All three intervention cohorts experienced, on average, a 33% reduction in anxiety level after the intervention and prior to their IVI. While there was a trend toward lower post-intervention anxiety among those in the music cohort compared to controls, this difference did not reach statistical significance, nor was there a significant difference in pain or satisfaction across all groups, including controls. The number of prior intravitreal injections was mildly inversely correlated with pain in all subjects.

Conclusion: In this study, brief pre-injection music and meditation interventions demonstrated intra-cohort anxiolytic effects, though they did not prove statistically effective at reducing anxiety or influencing perceptions of pain or patient satisfaction compared to control. We encourage further investigation into non-invasive methods for reducing pain and anxiety, as well as enhancing the patient experience during ophthalmologic procedures.

## Introduction

While the practice of meditation is thought to have originated at least 4,500 years ago [1], it was not until the mid-20th century that research began to shed light on its powerful psychological and neurophysiological effects [2-3]. Since then, meditation has been shown to reduce cortisol levels [4], result in reliable patterns of activation and deactivation on functional neuroimaging [5], and reduce anxiety, depression, and pain [6]. More recently, within the field of ophthalmology, meditation has been suggested to shorten central serous chorioretinopathy (CSR) episodes [7], and music has proven to reduce blood pressure, heart rate, pain, and anxiety during cataract surgery [8], as well as anxiety with intravitreal injections (IVIs) [9,10].

IVIs are the most common procedures performed by vitreoretinal specialists and are a cause of both patient anxiety and, in some cases, discomfort. These factors may influence patient satisfaction, treatment compliance, and, conceivably, by proxy, visual outcomes. To date, we have found no research in current literature on the effects of guided or non-guided, non-music-based meditation on the patient's experience with IVIs. Determining easy, non-invasive interventions that may mitigate the adverse psychological effects of IVIs may provide physicians with tools to better care for patients and optimize their experience and outcomes. The purpose of this study was to assess whether brief pre-injection exposure to spoken/guided meditation, nature sounds, or music could reduce anxiety and pain and improve satisfaction with IVIs.

## Materials and methods

This randomized controlled trial (RCT) was approved by the University of California, Los Angeles (UCLA) Institutional Review Board (IRB#19-000906) and carried out at the UCLA Jules Stein Eye Institute. The inclusion criteria included English-speaking outpatients who received IVIs within the Retina Division at the UCLA Jules Stein Eye Institute. The exclusion criteria included prior enrollment in the study. Patients were informed about the purpose of the study, and written informed consent was obtained from all participants prior to the commencement of the study. Patients who did not wish to participate were excluded from the study. Consecutive sampling was conducted from January 2021 to May 2022, during which 121 patients were selected for inclusion. The sample size was selected based on time and resource constraints.

Using a cyclic allocation based on the order of enrollment, patients were assigned to one of four cohorts: spoken/guided meditation (n = 31), nature sounds (n = 30), music (classical or jazz, n = 30), and control (n = 30). Patients in the first three cohorts were handed an iPad and headphones and instructed to listen to a preselected soundtrack for three to five minutes prior to receiving their IVI. The first cohort listened to a spoken/guided meditation soundtrack in the English language, the second cohort listened to a nature sounds soundtrack (their choice of relaxing river or forest sounds), and the third cohort listened to a music soundtrack (their choice of jazz or classical). Controls did not participate in an intervention prior to their IVI. The exact time between intervention and IVI varied depending on the injecting physician's availability, although the vast majority of these intervals were under 15-30 minutes. Patients were asked to rate their level of anxiety using a horizontal visual analog scale (VAS) labeled with integer values ranging from 0 (calm) to 10 (anxious) at three time points: pre-intervention, post-intervention, and post-IVI. In controls, subjective anxiety levels were recorded only before and after their IVI. The VAS for anxiety has been validated as a reliable indicator of preoperative and postoperative anxiety [11,12].

IVIs were completed by one of 12 injecting physicians under topical anesthesia, with or without subconjunctival lidocaine (SCL) (0.5 mL lidocaine PF 2% solution), the latter administered according to patient and physician preference and practice pattern. Topical anesthesia was achieved with one to two drops of 0.5% proparacaine solution and/or 0.5% tetracaine solution, sometimes in combination with topical lidocaine, as preferred by the patient and physician. Following the IVI, subjective pain and satisfaction were also recorded in all cohorts. Patients were asked if they experienced pain, discomfort, or burning during the injection. If yes, they were asked to indicate how much pain they experienced during the injection using a horizontal VAS labeled with integer values ranging from 0 (no pain) to 10 (worst pain of their life). Patients were also asked to rate their experience with their injecting physician using a horizontal VAS labeled with integer values ranging from 0 (worst experience) to 10 (best experience).

Patients were oriented to the study survey by a member of the research team, provided guidance as needed, offered privacy while completing the survey, and subsequently instructed to submit the completed survey to a designated box. Other pertinent variables, including patient demographics, treatment indication, use of SCL, and the number of prior IVIs received at our institution, were obtained through a retrospective chart review.

All statistical analyses were performed using SAS version 9.4 (SAS Institute, Inc., Cary, NC). Descriptive statistics were calculated for all study variables. Statistical differences in categorical variables across treatment groups were compared using Fisher's exact test. Paired differences in continuous outcome variables before and after intervention within cohorts were assessed using the Wilcoxon signed-rank test, while the differences in continuous variables across treatment groups were compared using Kruskal-Wallis tests. P values < 0.05 were considered statistically significant for all comparisons.

## Results

Descriptive analysis of the study population

A total of 121 patients were included in this study. The mean patient age was 74.6 ± 13.4, and 56.2% of patients (68/121) were female (Table 1). The most common indications for treatment included age-related macular degeneration (57.8%), retinal vein occlusion (15.7%), and diabetic macular edema (13.2%). Nearly two-thirds of subjects (77/121) received SCL prior to their IVI. The number of prior IVIs ranged from 0 to 160, with a mean of 38.0. There was no statistically significant difference in SCL use rates (p = 0.627) or number of previous IVIs (p = 0.105) between participants in the four cohorts. Additionally, there was no statistically significant difference in any outcome stratified by age or SCL use.

**Table 1:** Patient Characteristics SD = standard deviation; AMD = age-related macular degeneration; RVO = retinal vein occlusion (branch or central); DME = diabetic macular edema; CNV = choroidal neovascularization; Other CME = cystoid macular edema not from RVO or radiation; VRL = vitreoretinal lymphoma

Characteristic	Guided Meditation	Music	Nature Sounds	Control	Total
N	31	30	30	30	121
Age (mean ± SD)	73.0 ± 13.2	78.7 ± 13.7	68.6 ± 13.8	78.0 ± 11.0	74.6 ± 13.4
Gender (N (Col Pct))					
Male	14 (45.2%)	13 (43.3%)	11 (36.7%)	15 (50.0%)	53 (43.8%)
Female	17 (54.8%)	17 (56.7%)	19 (63.3%)	15 (50.0%)	68 (56.2%)
Indication (N (Col Pct))					
AMD	15 (48.4%)	12 (40.0%)	3 (10.0%)	16 (53.3%)	46 (38.0%)
AMD & CNV	5 (16.1%)	7 (23.3%)	8 (26.7%)	4 (13.3%)	24 (19.8%)
RVO	6 (19.4%)	4 (13.3%)	5 (16.7%)	4 (13.3%)	19 (15.7%)
DME	3 (9.7%)	4 (13.3%)	2 (6.7%)	4 (13.3%)	13 (10.7%)
CNV	2 (6.5%)	1 (3.3%)	3 (10.0%)	0	6 (5.0%)
Endophthalmitis	0	0	2 (6.7%)	1 (3.3%)	3 (2.5%)
Other CME	0	1 (3.3%)	2 (6.7%)	0	3 (2.5%)
DME & PDR	0	0	2 (6.7%)	1 (3.3%)	3 (2.5%)
Radiation	0	0	2 (6.7%)	0	2 (1.7%)
VRL	0	0	1 (3.3%)	0	1 (0.8%)
Retinal edema	0	1 (3.3%)	0	0	1 (0.8%)
Anesthetic (N (Col Pct))					
Topical only	13 (41.9%)	12 (40.0%)	11 (36.7%)	8 (26.7%)	44 (36.4%)
Subconjunctival lidocaine	18 (58.0%)	18 (60.0%)	19 (63.3%)	22 (73.3%)	77 (63.6%)
Number of previous injections (mean, range)	40.1, 0–138	49.5, 2–153	32.3, 0–152	30.1, 0–160	38.0, 0–160

The effect of intervention on anxiety

**Figure 1:**
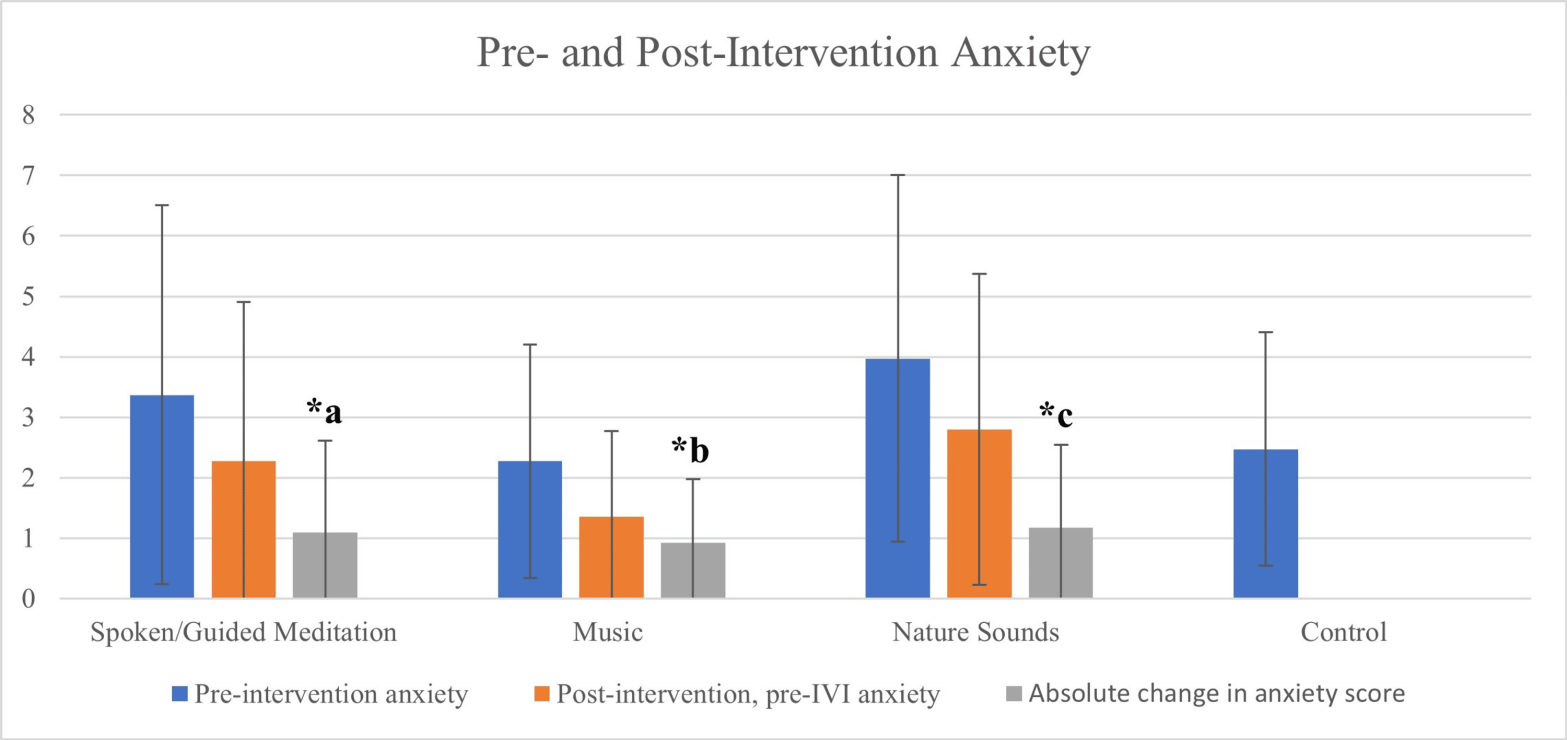
The Effect of Intervention on Anxiety Scores Prior to Intravitreal Injection (IVI) All intervention cohorts experienced, on average, a 33% relative reduction in anxiety levels after intervention prior to their IVI (a = -1.10 ± 1.51, p < 0.001; b = -0.92 ± 1.05, p < 0.001; c = -1.17 ± 1.37, p < 0.001). The error bars represent the standard deviation of the mean. There was no significant difference in pre-intervention anxiety scores between cohorts: spoken/guided meditation (3.37 ± 3.13), music (2.27 ± 1.93), nature sounds (3.97 ± 3.03), and controls (2.47 ± 1.93) (p = 0.133). Additionally, there was no significant difference in post-intervention, pre-IVI anxiety scores between the cohorts: spoken/guided meditation (2.27 ± 2.63), music (1.35 ± 1.42), nature sounds (2.80 ± 2.57), and controls (using the pre-intervention anxiety score) (p = 0.086).

All intervention cohorts experienced, on average, a 33% relative reduction in anxiety level post-intervention prior to their IVI (p < 0.001) (Table 2, Figure 1). All intervention cohorts experienced similar decreases in post-intervention, pre-injection anxiety, with a mean anxiety score change of -1.06 (p < 0.001, 95% CI (-1.33, -0.79)). The spoken/guided meditation cohort had a mean anxiety score change of -1.10 (p < 0.001, 95% CI (-1.65, -0.54)), the music cohort had a mean anxiety score change of -0.92 (p < 0.001, 95% CI (-1.31, -0.52)), and the nature sounds cohort had a mean anxiety score change of -1.17 (p < 0.001, 95% CI (-1.68, -0.66)). The mean change in pre- compared to post-intervention anxiety was greater in women than in men (female = -1.25, male = -0.76, p = 0.045).

**Table 2:** Intervention Effect on the Perception of Anxiety, Pain, and Satisfaction * Pre-intervention anxiety score was used for calculations

Variable	Guided Meditation (N=31)	Music (N=30)	Nature Sounds (N=30)	Control (N=30)	P-value (Across All Four Groups)	Combined Intervention Cohort (N=91)	P-value (Between the Combined Intervention Cohort and Control)
Before injection							
Pre-intervention anxiety	3.37 ± 3.13	2.27 ± 1.93	3.97 ± 3.03	2.47 ± 1.93	p=0.133	3.20 ± 2.82	p=0.371
Post-intervention (before IVI) anxiety	2.27 ± 2.63	1.35 ± 1.42	2.80 ± 2.57	- *	p=0.086	2.14 ± 2.33	p=0.175
Change in anxiety score (pre- vs. post-intervention, pre-IVI)	-1.10 ± 1.51 (p<0.001)	-0.92 ± 1.05 (p<0.001)	-1.17 ± 1.37 (p<0.001)	-	-	-1.06 ± 1.32 (p<0.001)	-
After injection							
Post-IVI anxiety	2.48 ± 2.76	1.90 ± 2.01	2.93 ± 2.75	2.57 ± 2.47	p=0.566	2.44 ± 2.54	p=0.606
Change in anxiety score (pre-intervention vs. post-IVI)	-0.89 ± 2.24 (p=0.024)	-0.37 ± 1.67 (p=0.243)	-1.03 ± 1.73 (p=0.002)	0.10 ± 1.75	p=0.081	-0.76 ± 1.90 (p<0.001)	p=0.028
Pain	1.68 ± 2.51	1.52 ± 2.07	1.48 ± 1.88	2.32 ± 2.93	p=0.702	1.56 ± 2.15	p=0.279
Satisfaction	9.42 ± 1.20	9.30 ± 1.76	9.43 ± 1.22	9.57 ± 1.30	p=0.661	9.38 ± 1.40	p=0.215

Pre-intervention, there was no statistically significant difference in baseline anxiety levels across all four cohorts (p = 0.133). Post-intervention, there was no statistically significant difference in anxiety levels between the four cohorts (p = 0.086), nor between the combined intervention cohort and control (p = 0.175). There was, however, a trend toward reduced post-intervention, pre-IVI anxiety level in the music group compared to the other cohorts, including controls, although this did not reach statistical significance (p = 0.086).

Post-IVI, there was no statistically significant difference in anxiety scores between the four cohorts (p = 0.566), nor between the combined intervention cohort and control (p = 0.606). All intervention cohorts experienced relative reductions in anxiety post-IVI compared to their baseline pre-intervention anxiety scores, with a mean anxiety score change of -0.76 ± 1.90 (p < 0.001) (Table 2). There was no statistically significant difference in the pre-intervention vs. post-IVI mean anxiety score change between the four cohorts (p = 0.081). There was, however, a mildly significant difference in pre-intervention vs. post-IVI mean anxiety score change between the combined intervention cohort and control (p = 0.028).

The effect of intervention on the perception of pain and satisfaction

**Figure 2:**
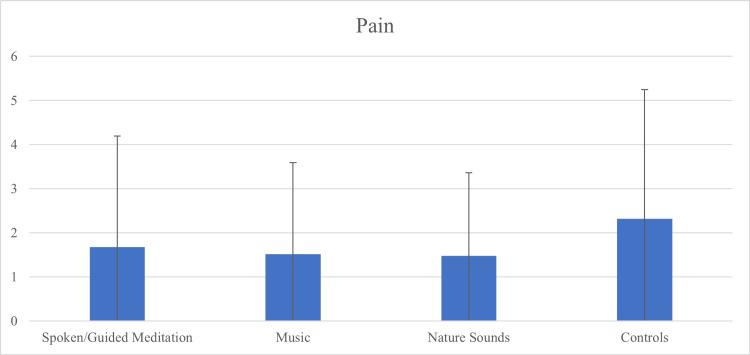
The Effect of Intervention on Pain Scores Post-Intravitreal Injection (IVI) There was no significant difference in pain scores between the combined intervention cohort and control (p = 0.279) or between cohorts: spoken/guided meditation (1.68 ± 2.51), music (1.52 ± 2.07), nature sounds (1.48 ± 1.88), and control (2.32 ± 2.93) (p = 0.702).

Following IVI, there was no statistically significant difference in pain scores between all four cohorts (p = 0.702) (Table 2, Figure 2), nor between the combined intervention cohorts and control (p = 0.279). In contrast, an increased number of prior IVIs was associated mildly with decreased subjective pain among all participants (r = -0.20, p = 0.030). No similar correlations between the number of previous IVIs and anxiety levels or satisfaction rates were observed.

**Figure 3:**
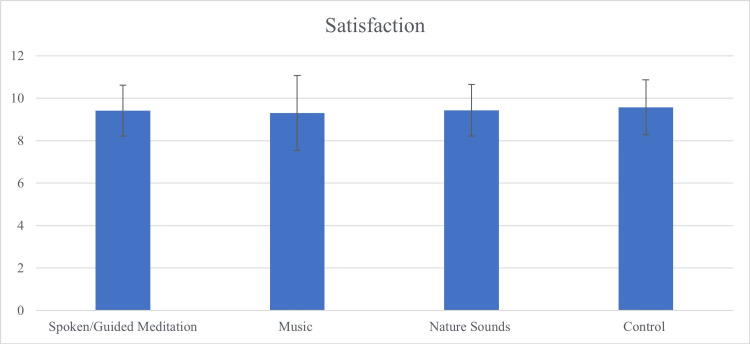
The Effect of Intervention on Satisfaction Scores Post-Intravitreal Injection (IVI) There was no significant difference in satisfaction between the combined intervention cohort and control (p = 0.215) or between cohorts: guided meditation (9.42 ± 1.20), music (9.30 ± 1.76), nature sounds (9.43 ± 1.22), and control (9.57 ± 1.30) (p = 0.661).

Finally, there was no statistically significant difference in satisfaction scores between all four cohorts (p = 0.661) (Table 2, Figure 3), nor between the combined intervention cohorts and control (p = 0.215).

## Discussion

To our knowledge, this is the first RCT to evaluate the effects of guided or non-guided, non-music-based meditation during IVIs. IVI of pharmacological agents is a mainstay of treatment for common retinal pathologies, including age-related macular degeneration, vascular occlusions, diabetic retinopathy, endophthalmitis, and cystoid macular edema [13]. The pain and discomfort associated with these injections may reduce compliance, a crucial component of long-term ophthalmologic care for diseases that require recurrent injections [14,15]. Further, Segal et al. demonstrated a relationship between preprocedural anxiety and pain reported during IVIs [16]. Thus, it may be valuable to identify inexpensive, non-invasive methods for reducing pain and anxiety during IVIs to promote compliance and improve ophthalmologic outcomes. The objective of this study was to determine whether brief interventions, including guided meditation, music, or nature sounds, may be effective strategies for alleviating discomfort during IVIs.

A small number of studies have investigated alternative methods for reducing pain and discomfort experienced by patients during IVIs. Gomez et al. surveyed 128 patients and found that the presence of an extra staff member during the injection, use of a neck pillow, provision of a verbal warning prior to injection, and completion of bilateral eye injections on the same day were indicated by patients as favorable strategies for improving comfort during IVIs [17]. Another study found that patient anxiety levels significantly reduced when their hand was held during IVI and that anxiety did not lessen with repeated injections [18]. We similarly found no correlation between anxiety levels and the number of previous IVIs.

Other studies have explored the utility of music therapy. Bradt et al. found that music interventions may have beneficial effects on anxiety, pain, fatigue, and quality of life in people with cancer [19]. Within the field of ophthalmology, randomized trials have shown that music is an effective anxiolytic and may reduce the perception of pain during cataract surgery, both subjectively with reduction in patient-reported anxiety index scores and visual analog pain scale, and objectively with reduction in heart rate and blood pressure reflecting downregulation of the sympathetic nervous system [8,20-22]. Similarly, Chen et al. demonstrated a significant decrease in patient anxiety levels while undergoing IVIs when exposed to classical music before and during the procedure, compared to controls without a music intervention [10]. Brosh et al. found significantly lower post-injection subjective anxiety levels in patients undergoing IVIs who were allowed their unlimited choice in music before and during the procedure, compared to patients for whom the physician chose the music and also compared to controls [9]. They also reported no statistically significant difference in subjective pain between cohorts.

In our study, average pre-intervention anxiety levels across cohorts (2.27-3.97 out of 10) were comparable to those reported by Brosh et al. (2.7-3.7). While patients in the present study randomized to the music cohort were allowed to choose from a pre-selected set of jazz or classical tracks, they were not offered an unlimited choice in music, as was the case in the study by Brosh et al. This expanded selection appeared to significantly differentially lower anxiety in the aforementioned study compared to a lack of music or non-preferred/physician-chosen music. We noted a trend of lower post-intervention, pre-IVI anxiety in the music compared to the other cohorts, including controls. Although this did not reach statistical significance (p = 0.086), possibly due to the limited sample size, it is a signal worthy of further investigation in future studies.

Notably, subjective anxiety prior to IVIs has been previously found to correlate with patients' perception of pain, as measured on VAS (0-10) [16]. While intuition and our knowledge of the sympathetic nervous system's role in states of elevated stress, anxiety, and hyperawareness of external stimuli might support this relationship, our data did not yield corresponding statistically significant correlations between patients' subjective anxiety and pain levels, nor did the interventions significantly influence pain perception. Effective local anesthesia may have masked such relationships, given the low reported pain levels (Figure 3). However, the mild inverse relationship between prior IVI experience and pain perception suggests that cumulative procedural experience, informing patients' expectations, influences actual subjective comfort, regardless of presumably sufficient local anesthesia.

We acknowledge several limitations in the present study. A total of 12 treating physicians were included, whose exact IVI techniques may differ, although randomization should minimize this effect on outcomes. The exact time between intervention and IVI varied, with longer wait times potentially attenuating the benefits of intervention or allowing for influence from external factors, although the vast majority of these intervals were under 15-30 minutes. While there was no significant difference in the number of participants who utilized subconjunctival lidocaine injections between the four cohorts, we do recognize that the use of SCL may affect patients' pain perceptions compared to topical anesthetics.

Additionally, patients' prior experience levels with meditation were not evaluated. As such, it is possible that a differential influence or compounding effect of habitual meditative practice might have gone unrecognized. As mentioned, patients were given the option and encouraged to privately complete the surveys to minimize the Hawthorne effect, although many preferred to complete the survey with some assistance, possibly eliciting this effect (i.e., survey response bias may be present if subjects felt their responses were inadequately blinded). Participants were also informed of the purpose of the study during enrollment, which may have influenced the subjective reporting of anxiety scores. Furthermore, other factors that were not evaluated, such as ophthalmologic comorbidities or sociodemographic characteristics, may have also influenced participants' IVI experience.

An intervention duration of three to five minutes was chosen because it allows for a consistent continuation of meditation, regardless of whether the subject is new to meditation or more experienced. Multiple studies have supported the benefits of brief (i.e., five minutes or less) breathwork and mindfulness meditations on improving mood and reducing anxiety [23,24]. However, it is possible that a longer intervention duration or continuing the meditative intervention during the IVI might have conferred a differential benefit. Further, the brief spoken/guided meditation, nature sounds, and music soundtracks selected for this study have not been previously validated. Simple 0-10 analog scales were chosen for their efficiency and simplicity to minimize any study participation contribution to patient anxiety; however, less data granularity might decrease the ability to discern subtle differences between cohorts. Future studies may benefit from utilizing more extensive and in-depth instruments such as the Spielberger State-Trait Anxiety Inventory (STAI-S), a validated 20-question instrument used to measure anxiety with a score ranging from 20 to 80 [25]. While our study had one of the largest enrollments to date in this realm, it is possible that the study was underpowered, and larger enrollment numbers may be required to detect more subtle differences between cohorts for the studied outcomes.

## Conclusions

In conclusion, the brief one-time music and meditation interventions in this study did not significantly impact patient satisfaction, and they did not, individually, significantly impact anxiety levels compared to controls. No effect was observed on pain perception, except for an inverse correlation with the number of prior IVIs and subjects' pain levels, suggesting that expectations based on prior experience may effectively confer a subjective analgesic effect. The anxiolytic signals of the interventions studied, however, within each cohort, as well as the statistically significant difference in mean anxiety levels pre-intervention vs. post-IVI between combined intervention cohorts compared to controls, suggest that further investigation is warranted into similar inexpensive, non-invasive methods that may reduce anxiety and enhance the patient experience during ophthalmologic procedures.
